# Women’s Experience of Disrespect and Abuse during Institutional Delivery in Biratnagar, Nepal

**DOI:** 10.3390/ijerph18189612

**Published:** 2021-09-12

**Authors:** Narayani Paudel Ghimire, Sunil Kumar Joshi, Pranab Dahal, Katarina Swahnberg

**Affiliations:** 1 Department of Nursing, Kathmandu Medical College, Kathmandu 44600, Nepal; 2 Head of Department, Community Medicine, Kathmandu Medical College, Kathmandu 44600, Nepal; drsunilkumarjoshi@gmail.com; 3 Department of Health and Caring Sciences, Linnaeus University, 39182 Kalmar, Sweden; pranab.dahal@lnu.se (P.D.); katarina.swahnberg@lnu.se (K.S.)

**Keywords:** abuse, disrespect, institutional delivery, obstetric violence, Nepal

## Abstract

Worldwide, a large number of women experience disrespectful and abusive behavior from care providers during childbirth. This violates the rights of women to attain respectful care. This study aimed to find out the women’s experience of disrespect and abuse during institutional delivery. A cross-sectional study was conducted in two hospitals of Morang district situated in eastern Nepal. Two hundred eighteen women from a public hospital and 109 women from a private hospital (*N* = 327) with normal vaginal delivery were selected purposively for this study. Data were collected through face-to-face interviews using a structured questionnaire based on the Disrespectful and Abusive Scale by Bowser and Hill. All women had experienced at least one type of disrespect and/or abuse during labor and delivery, most common being non-consented care (100%), non-dignified care (72%), and non-confidential care (66.6%), respectively. Discriminatory care and physical abuse were experienced by 32.33% and 13.23%, respectively. Ethnicity, religion, place of delivery, and numbers of living children were the main predictors of reporting disrespect and abuse. Overall, the occurrence of disrespect and abuse during institutional delivery was found to be very high.

## 1. Introduction

The provision of respectful and satisfactory maternity care is essential for promoting timely care-seeking behavior and ultimately ensuring the health and well-being of mothers and their babies. Disrespectful and abusive behavior from health care providers has been recognized as one of the barriers to seeking timely maternity health services [1]. Every woman has the right to: be free from harm and ill treatment; information, informed consent, and refusal of treatment, respect for her choices and preferences, including the right to choose her birth companion; privacy and confidentiality. She also has the right to be treated with dignity and respect, and to expect the following: equality; freedom from discrimination and equitable care; health care; and, to the highest attainable level of health, liberty, autonomy, self-determination, and freedom from coercion [2].

Disrespectful and abusive care includes impoliteness of care providers, inappropriate reprimands, shouting at the client, lack of empathy, refusal to assist, threatening clients for their non-compliance, and denying clients opportunities to choose or give an opinion on the care they are receiving [3]. Based on a comprehensive review, seven categories of disrespect and abuse in childbirth were identified by Browser and Hill: physical abuse, non-consented care, non-confidential care, non-dignified care, discrimination based on specific patient attributes, abandonment of care, and detention in facilities [4].

Globally, many women experience mistreatment during childbirth, including abusive anddisrespectful care.Women aremistreatedacross all income levels and at alllocations. Previous studies conducted in various countries have shown that 27.2% to 98% of women experienced at least one type of disrespectful and abusive behavior from care providers during facility-based childbirth [5,6,7,8,9,10]. The mistreatmentranges fromneglect, poor communication, to the extreme: slapping, pinching, gags, threatening, and verbal abuse [11].

Maternal and child mortality rates are still high in Nepal. The recent maternal mortality ratio (MMR) is 186 deaths per 100,000 live births, neonatal mortality rate is 19.8deaths per 1000 live births, infant mortality rate is 25.6 deaths per 1000 live births, and child mortality rate is 30.8 deaths per 1000 live births [12]. Sustainable Development Goal 3 targets the reduction of maternal mortality rate (MMR) to less than 70 per 100,000 live births by 2030 [13]. Key to reducing maternal mortality is to promote institutional delivery rather than at home. However, women will avoid hospitals if they are mistreated [11].

White Ribbon Alliance claims that in Nepal, women in childbirth routinely experience disrespect and abuse in health facilities. The problem is pervasive and does not spare anyone, regardless of social, geographic, or economic background. The overall disrespect and abuse was reported by 70.1% of the women in a study conducted in Pokhara, Nepal [14]. This is not only a violation of human rights, but it discourages pregnant women from seeking healthcare that could save their lives [15]. In October 2018, the Government of Nepal adopted the Safe Motherhood and Reproductive Health Rights Act. The legislation marks for the first time that respectful maternity care has been included in national legislation and paves the way for the provision of high-quality, respectful care for mothers and babies in public and private health facilities in the country [16]. The use of abusive behaviors against women during childbirth in delivery facilities has been the subject of numerous studies, predominately in developing countries [17]. It has been shown to have negative impacts on the overall quality of care and trust in health providers and systems and is associated with adverse maternal and neonatal outcomes [18].

There is a lack of adequate studies in Nepal that address the prevalence and factors associated with disrespect and abuse of women in facility-based childbirth. Disrespectful and abusive behaviors might be associated with, e.g., ethnicity, religion, and/or education level. Thus, this study was conducted to learn more about the women’s experiences and factors related to reporting disrespect and abuse during childbirth as a quest for respectful maternity care in Nepal.

## 2. Materials and Methods

### 2.1. Design and Setting

A cross-sectional study was conducted in one public and one private hospital in Biratnagar, Morang district, situated in Province 1, Nepal. Public hospitals in Nepal have low resources compared to private hospitals. Maternal and child health services are free of cost in public hospitals of Nepal. Besides, women receive transportation costs when they deliver in a health facility whereas, women must pay for the services in private hospitals. The professional background of the care providers is similar in both hospitals. Generally, normal deliveries in both hospitals are conducted by nurses. One of the reasons for selecting these sites was to compare the women’s experience of disrespect and abuse in two different settings. Moreover, the study site is a home for populations from various socio-demographic characteristics and backgrounds. In addition, large numbers of women from the adjoining region visit public and private hospitals located in the study site, seeking maternal health services. Thus, these places have been selected purposefully to fulfill the objectives of the study.

### 2.2. Study Population and Sample Size

Women admitted to the postnatal ward after normal vaginal deliveries were included in the study. Based on the prevalence of disrespect and abuse in a previous study performed in Gujrat, Pakistan [1], prevalence (P) 27.2% with 95% confidence intervals, the calculated sample size was 303. Adding on an 8% non-response rate, the final sample size was estimated to be 327 women; 218 women from a public hospital and 109 women from a private hospital were selected for the study using a consecutive sampling technique. Every consenting woman having normal delivery was consecutively included till the required sample size was met. Women who had undergone childbirth through caesarean section were excluded from the study. As vaginal deliveries in these hospitals are mostly conducted by nurses and caesarean sections are performed by obstetricians, the reason behind excluding caesarean birth was to identify the nurses’ behavior towards women during labor and delivery.

### 2.3. Data Collection Tool

A structured questionnaire was designed based on the Disrespect and Abuse (D & A) scale according to Bowser and Hill’s landscape evidence review. The authors adopted this scale as it has comprehensive points to find out the abuse faced by women during facility-based childbirth. The D & A scale includes seven categories of disrespect and abuse in childbirth: (1) physical abuse, (2) non-consented care, (3) non-confidential care, (4) non-dignified care, (5) discrimination based on specific patient attributes, (6) abandonment of care, and (7) detention in facilities. These categories of disrespect and abuse during facility-based childbirth were adopted from the above-mentioned scale and 28 items under these categories were developed based on extensive literature review. Items were categorized into binary choice: yes/no to find out whether the women experienced disrespect and abuse or not. All the items are presented in Table 1. The tool was translated into Nepali language and back translation to English was performed. Pretesting of the tool was conducted among 10% of the sample size to maintain the validity of the tool. Cronbach’s alpha was 0.82 in the pretest data to find out the reliability of the tool.

### 2.4. Data Collection Procedure

Data collection was performed from September to October 2020. The first author collected the data using the face-to-face interview technique. Different understanding and expectations of non-confidential care, non-dignified care, discrimination, and abandonment of care might occur.So, the author asked the questions of the respondents in local and understandable language. All interviews were conducted in the postnatal ward on the day of discharge in a separate room to maintain privacy and confidentiality.

### 2.5. Outcome Measures

The outcome variable for this study was experiences of disrespect and abuse during labor and delivery based on 28 indicators classified into 7 categories, Table 1.

Independent variables were: socio-demographic characteristics (age, ethnicity, religion, education, and occupation) and obstetric characteristics (parity, number of living children, and place of delivery).

### 2.6. Data Analysis

Collected data were coded, entered, and analyzed using a statistical package for social science (SPSS) version 20. Descriptive statistics such as frequency, percentage, mean, and standard deviation were used to describe the socio-demographic and obstetric characteristics of the participants as well as prevalence of disrespect and abuse in childbirth. Women who reported experiencing one or more subcategories of disrespect were included in the overall prevalence measure. The sum score of experiencing D&A for each woman was calculated (varying from 0 to 28) and this variable was used as the outcome variable. Mann–Whitney U and Kruskal–Wallis tests were performed to identify the factors associated with experience of disrespect and abuse. Multiple linear regressions were calculated to identify the strength of association. Statistical significance level was set at 95% (*p*-value < 0.05).

### 2.7. Ethical Considerations

For the ethical consideration, a proposal was submitted to Nepal Health Research Council (NHRC), and ethical clearance was obtained from the Ethical Review Board of NHRC (Ref. No. 2088). A proposal was also submitted to concerned authorities of both the hospitals and written permission for data collection was obtained from both the hospitals. To maintain anonymity of the hospitals, the name of the hospitals has not been disclosed anywhere. Each respondent received an explanation about the purpose of the study and written informed consent was taken from each respondent before the interview.

## 3. Results

Sociodemographic information of the respondents is presented in Table 2. More than half of the respondents (60.1%) from the public hospital and around half of the respondents (47.7%) from the private hospital belonged to the age group 20–25 years, with mean age 22 and 23.8 years, respectively (range 17–38 years). The majority of the respondents (63.3%) from the public hospital and more than half of the respondents (52.3%) from the private hospital were from the Madhesi ethnic group. Most of the respondents (90.8% and 92.7%, respectively) from the public hospital and the private hospital practiced the Hindu religion, only 3.2% of women from the public hospital and 10.1% of women from the private hospital had obtained bachelor’s or above level of education, and most of the respondents (83.5% and 79.8%, respectively) from both hospitals were housewives. In terms of obstetric information, the majority of the women (around 55%) were primiparous and a majority of the women (57.8%) from both the hospitals had only one living child. Distribution of respondents according to age, ethnicity, and education was significantly different between the two hospitals (*p* ≤ 0.05).

Table 3 depicts the disrespect and abuse experienced by women during labor and delivery. All women reported that they received non-consented care. Episiotomy given without adequate anesthesia, 54.1%, and suturing without adequate anesthesia, 63%, were also commonly reported. Overall, physical abuse like being bitten, slapped, and/or other kinds of rough touch was experienced by 13% of the women. All women from the public hospital reported that privacy was not maintained during physical examination, and all women from the private hospital felt that the number of staff around them was illogical during their delivery. Similarly, the majority of women (72.2%) reported that providers did not speak politely and almost all (97.9%) reported that they were not encouraged to ask questions even when needed. Many women (58.7%) faced language barriers and around one fourth (24.8%) reported discrimination based on sociocultural attributes, such as ethnicity, religion, and/or education. None of the women were allowed to keep their husband with them during labor, 99.4% of women were not attended to immediately by providers when needed, 96% of women were not encouraged to call providers when needed, and more than one third (35%) of the women felt alone and unattended during labor.

Mann–Whitney U and Kruskal–Wallis were performed to identify the factors associated with disrespect and abuse (Table 4). Disrespectful and abusive behaviors experienced by women during labor and delivery were significantly associated with ethnicity, religion, level of education, number of living children, and place of delivery (*p* ≤ 0.05).Multiple linear regression analysis was performed to identify the strength of association (Table 5). It revealed that women’s experience of disrespect and abuse during labor and childbirth was significantly associated with Madhesi ethnicity, Aadibasi-Terai and Muslim caste (*β* = 2.459, 1.593, and 3.278, respectively), Muslim religion (*β* = 1.095), having delivered in a private hospital (*β* = 2.285), and having two or more living children (*β* = 0.904) with *p*-value < 0.05. Despite this, most women who want to have more children reported intending to deliver again at the same facility as they have no other better choice.

## 4. Discussion

Providing respectful maternity care and increasing institutional delivery are the focus of health programs of the Nepal government. Promoting respectful maternity care during childbirth is a vehicle to utilize maternal health care and prevent maternal and child morbidity and mortality rate. Disrespectful and abusive behaviors during childbirth and maternity care have been documented throughout the world, making this truly a global agenda. This study aimed to find out the prevalence of disrespectful and abusive behavior during institutional delivery.

In this study, all women reported at least one category of disrespect and abuse during labor and delivery, the most common being non-consented care, non-confidential care, and non-dignified care. Similar studies conducted in Pakistan and Nigeria also found that almost all women (99.7% and 98%, respectively) experienced at least one type of abusive behavior during childbirth [5,7]. However, the overall disrespect and abuse reported during facility-based childbirth was 70.1% in a study conducted in Pokhara, Nepal [14].

In the present study, 100% of women experienced non-consented care, 72.2% of women experienced non-dignified care, and 66.6% of women experienced non-confidential care, which was similar to a study conducted in Gujrat, Pakistan in which it is mentioned that the most commonly experienced disrespect and abuse was non-consented care and lack of informed choice (99.7%) followed by non-confidential care (58.6%), and non-dignified care (45.6%) [1]. Findings were also similar to studies conducted in Nigeria, Ethiopia and Pakistan, in which non-consented care and lack of information, lack of privacy, and non-dignified care were the most common instances of disrespect and abuse [5,8].

In our study, abandonment of care (neglected) was experienced by 35.6% which was also consistent with the findings of studies conducted in Nigeria and Pakistan in which 29.1% and 32% of women reported experience of abandonment of care, respectively [7,8]. In this study, discriminated care based on socio-cultural attribution (e.g. Madhesi caste, Muslim religion) was reported by 25%. This finding is similar to the study conducted in Nigeria where 20% of women reported discrimination on the basis of ethnicity, low social class, and young age [7].

Overall, physical abuse such as beating, slapping, pinching, and rough touch was reported by 12.23% of women in this study which was almost similar to studies conducted in Pakistan and Ethiopia where 15% and 13.6% of women had reported physical abuse, respectively [8,17]. However, a higher number of women (18.7%) reported physical abuse in a study conducted in Kathmandu, Nepal [19]. This could be due to differences in awareness and perceptions of respectful maternity care in different settings. Detentions in health facilities have been shown in various previous studies [8,17] but it was almost not reported in our study. This may be due to the difference in health policies of hospitals. Maternity services are free of cost in the government hospital of Nepal.

Disrespect and abuse during labor and delivery were significantly associated with ethnicity, religion, level of education, number of living children, and place of delivery on univariate analysis. The finding was consistent with studies conducted in Pakistan and southern Mozambique and in which ethnicity, educational level, and parity were the predictors of disrespect and abuse [8,9]. However, on multiple linear regression, disrespect and abuse were significantly associated with Aadibasi-Terai and Muslim ethnicity (*β* = 2.45 and 3.27, respectively), delivered in private hospital (*β* = 2.28). In this study, women with more than one living child were more likely (*β* = 0.904 with 95% CI = 0.452, 1.356)to report disrespect and abuse compared to women with only one child. This is in contrast with a study conducted in southwest Ethiopia in which parity two or above women were nearly 72% less likely (AOR: 0.283; 95%CI: (0.067, 0.762)) to report disrespect and abuse compared to parity one woman [10]. The difference may be due to ignorance of respectful maternity care among primipara rather than multipara. In our study, women who delivered their child in a private hospital were more than two times more likely (*β* = 2.285 with 95% CI = 1.806, 2.763) to experience disrespect and abuse. In contrast, a study conducted in Pakistan found that the risk of reporting disrespect and abuse was twice in public health facilities as compared to private [5]. The difference may be due to deliveries conducted by skilled birth attendant trainees in the public hospital, as skilled birth attendant training was going on in the public hospital during the data collection period. Moreover, there might be high expectations of women from the private hospitals or it may be due to increased awareness of women’s right to obtain respectful maternity care.

However, there are minimal studies related to women’s experience of disrespect and abuse during institutional delivery in Nepal. The findings of the study might be the basis for intervention to enhance respectful maternity services. The findings of the study might be useful to maternity care providers. The limitations of this study are a small sample size and non-probability sampling. Since this is a sensitive and subjective issue, the response might be under- or over-reported. The findings of the study are based on women’s reports only. Different understanding and expectations of non-confidential care, non-dignified care, discrimination, and abandonment of care might occur. Cultural and social practices shape self-understanding and how women understand themselves as being neglected or discriminated against. These value-loaded concepts could also have been investigated with qualitative research methods and ethnography in order to understand these women’s expectations, thinking, and needs. Thus, further studies among women as well as health care workers, using qualitative research design, are planned.

## 5. Conclusions

Findings of the study revealed that the prevalence of disrespect and abuse during labor and delivery is high in institutional delivery both in public as well as private hospitals. Disrespect and abuse during labor and delivery were significantly associated with ethnicity, religion, level of education, number of living children, and place of delivery. Despite this, most women who want to have more children reported intending to deliver again at the same facility, as they have no other better choice. Thus, measures to enhance respectful maternity care are essential. To reduce the disrespect and abuse of women during institutional delivery, respectful maternity care components should be incorporated in the nursing and medical education curricula so that the care providers will be prepared for this from the pre-service period. Guidelines on respectful maternity care should be formulated and communicated to all the maternity care providers.

## Figures and Tables

**Table 1:** Categories and items of disrespect and abuse during childbirth.

S N.	Categories and Items of D & A Scale	Yes	No
1	Physical abuse		
1.1	Used physical force		
1.2	Bitten, slapped, or pinched		
1.3	Touched roughly		
1.4	Episiotomy given or sutured without anesthesia		
1.5	Restrained or tied down during labor		
2	Non-consented care		
2.1	Provider did not introduce herself		
2.2	Provider did not explain procedure and expectations		
2.3	Provider did not give periodic updates on status and progress		
2.4	Provider did not obtain consent prior to procedure		
3	Non-confidential care		
3.1	Curtains and physical barriers were not used		
3.2	Drape or body covering was not used		
3.3	The number of staff members around was not logical (more than required)		
3.4	Provider disclosed my personal/medical history to third parties		
4	Non-dignified care		
4.1	Provider did not speak politely, truthfully, and promptly		
4.2	Provider made insults, threats, etc.		
4.3	Provider did not encourage me to ask questions		
4.4	Provider used abusive language		
5	Discriminatory care		
5.1	Provider used language difficult to understand		
5.2	Provider showed disrespect based on specific attribute (e.g., caste, religion)		
5.3	Provider did not demonstrate in a culturally appropriate way		
6	Abandonment of care		
6.1	Provider did not encourage me to call if needed		
6.2	Provider made me feel alone or unattended		
6.3	Provider did not come quickly when needed		
6.4	Baby was separated without medical indication		
6.5	My husband was not allowed to sit with me during labor		
7	Detention in facility		
7.1	Provider did not allow me to move during delivery		
7.2	Provider did not allow me to assume position of choice		
7.3	Detained me/ my baby due to bill not paid		

**Table 2:** Sociodemographic characteristics of respondents (*N* = 327).

	Public Hospital (*n* = 218) *n* (%)	Private Hospital (*n* = 109) *n* (%)	*p*-Value
Age (in years)			0.03
<20	25 (11.5)	14 (12.8)	
20–25	131 (60.1)	52 (47.7)	
25–30	55 (25.2)	32(29.4)	
>30	7 (3.2)	11 (10.1)	
Mean ± S.D	22.77 ± 3.37	23.84 ± 4.85	< 0.001
Ethnicity			0.003
Madhesi *	138 (63.3)	57 (52.3)	
Aadibasi-Terai	38 (17.4)	14 (12.8)	
Caste-Hill ^#^	22 (10.1)	30 (27.5)	
Muslim	20 (9.2)	8 (7.3)	
Religion			0.57
Hindu	98 (90.8)	101 (92.7)	
Muslim	20 (9.2)	8 (7.3)	
Education			0.02
Non-formal	60 (27.5)	18 (16.5)	
Primary level	38 (17.4)	15 (13.8)	
Secondary level	86 (39.4)	48 (44.0)	
Higher secondary level	27 (12.4)	17 (15.6)	
Bachelor and above	7 (3.2)	11 (10.1)	
Occupation			0.28
Housewife	182 (83.5)	87 (79.8)	
Business	20 (9.2)	16 (14.7)	
Laborer	16 (7.3)	6 (5.5)	
Parity			0.81
Primi	121 (55.5)	59 (54.1)	
Multi	97 (44.5)	50 (45.9)	
Living children			0.99
One	126 (57.8)	63 (57.8)	
Two	71 (32.6)	35 (32.1)	
Three or more	21 (9.6)	11 (10.1)	

* People of Indianancestryresiding in the Terai of Nepal. ^#^ Brahmin, Chhetri, Magar, Rai, Limbu, Damai, and Kami.

**Table 3:** Disrespect and Abuse during labor and delivery (*N* = 327).

Abusive Behavior Reported	Public Hospital (*n* = 218) *n* (%)	Private Hospital (*n* = 109) *n* (%)	*p*-Value
Physical Abuse			
Bitten, slapped, or pinched	15 (6.9)	17 (15.6)	0. 01
Touched roughly	22 (10.1)	18 (16.5)	0. 09
Episiotomy given without adequate anesthesia	146 (67.0)	31 (28.4)	<0.001
Sutured without adequate anesthesia	165 (75.7)	41 (37.6)	<0.001
Non-consented care			
Provider did not introduce herself	218 (100)	109 (100)	-
Did not explain about procedure	218 (100)	109 (100)-	-
Did not explain expectations	218 (100)	109 (100)	-
Did not give periodic updates	218 (100)	109 (100)	-
Did not obtain consent prior to procedure	218 (100)	109 (100)	-
Non-confidential care			
Curtains and physical barriers not used	218 (100)	0 (0.0)	-
Drape or body covering was not used	218 (100)	0 (0.0)	-
The numbers of staff around was not logical	0 (0.0)	109 (100)	-
Non-dignified care		
Did not speak politely	157 (72.0)	79 (72.5)	0.93
Made insults, threats, etc.	10 (4.6)	13 (11.9)	0.01
Did not encourage patient to ask questions	215 (98.6)	105 (96.3)	0.17
Used abusive language	16 (7.3)	5 (4.6)	0.33
Discriminatory care			
Used language difficult to understand	134 (61.5)	58 (53.2)	0.15
Showed disrespect based on specific attribute	26 (11.9)	55 (50.5)	<0.001
Did not demonstrate in culturally appropriate way	26 (11.9)	20 (18.3)	0.11
Abandonment of care		
Did not encourage patient to call if needed	217 (95.0)	107 (98.2)	0.16
Made patient feel alone or unattended	8 (3.7)	108 (99.1)	<0.001
Husband was not allowed to sit with patient during labor	218 (100)	109(100)	-
Provider did not come quickly when needed	216 (99.1)	109 (100)	-
Detention in health facility	0	0	-

**Table 4:** Factors associated with disrespect and abuse of women during childbirth (*N* = 327).

Independent Variables	*N*	Median Score	*p*-Value
Ethnicity			<0.001 ^#^
Madhesi	195	15	
Aadibasi-Terai	52	14	
Caste-Hill	52	13	
Muslim	28	16	
Religion			0.012 *
Hindu	299	15	
Muslim	28	16	
Education level			0.045 ^#^
Non-formal and primary	131	15	
Secondary	134	15	
Higher secondary and above	62	14	
Number of living children			0.012 *
One	189	15	
Two or more	138	14	
Place of delivery			<0.001 *
Public hospital	218	14	
Private hospital	109	16	

* Mann–Whitney U test, ^#^ Kruskal–Wallis. **Note**: Median score was calculated splitting the file on the basis ofeach sociodemographic variable.

**Table 5:** Multiple linear regression analysis for the factors (*N* = 327).

	Coefficient	95% CI
Ethnicity		
Madhesi	1	
Aadibasi-Terai	2.45	1.82, 3.09
Caste-Hill	1. 59	0.79, 2.38
Muslim	3.27	2.32, 4.22
Religion		
Hindu	1	
Muslim	1.09	1.06, 2.02
Education level		
Non-formal and primary	1	
Secondary	0.50	−0.14, 1.14
Higher secondary and above	0.30	−0.18, 0.79
Number of living children		
One	1	
Two or more	0.90	0.45, 1.35
Place of delivery		
Public hospital	1	
Private hospital	2.28	1.80, 2.76

## Data Availability

The raw data supporting the conclusions of this article will be made available by the authors upon reasonable request.
